# YAP1-NUTM1 Gene Fusion in Porocarcinoma of the External Auditory Canal

**DOI:** 10.1007/s12105-020-01173-9

**Published:** 2020-05-20

**Authors:** Abbas Agaimy, Lars Tögel, Florian Haller, Johannes Zenk, Joachim Hornung, Bruno Märkl

**Affiliations:** 1 Institute of Pathology, University Hospital, Erlangen, Germany; 2 Department of Otorhinolaryngology, Head and Neck Surgery, University Hospital, Augsburg, Germany; 3 Department of Otorhinolaryngology, Head and Neck Surgery, University Hospital, Erlangen, Germany; 4 Institute of Pathology and Molecular Diagnostics, University Hospital, Augsburg, Germany

**Keywords:** NUT carcinoma, NUTM1, YAP1, Midline carcinoma, Ear, Head and neck, Auditory canal

## Abstract

Gene fusions involving the *NUTM1* gene (*NUT*) represent defining genetic markers of a highly aggressive carcinoma type with predilection for the midline structures of children and young adults, hence the original description as NUT midline carcinoma. Recent studies have increasingly documented involvement of the *NUTM1* gene in the pathogenesis of other entities as well. We herein describe two cases of auditory canal carcinomas with features of porocarcinoma, both harboring a newly described *YAP1-NUTM1* gene fusion. Patients were males aged 28 and 82 years who presented with slowly growing lesions in the external auditory canal. Histologic examination showed monomorphic basaloid and squamoid cells arranged into organoid solid aggregates, nests, ducts, small cysts, and focal pseudocribriform pattern with variable mitotic activity, infiltrative growth, and focal squamous differentiation, particularly in the most superficial part of the tumor. Immunohistochemistry revealed consistent reactivity for CK5, p63 and SOX10 and diffuse aberrant expression of TP53. CK7 expression was limited to a few luminal ductal cells. The androgen receptor and S100 were negative. Next generation sequencing (TruSight RNA fusion panel, Illumina) revealed the same *YAP1-NUTM1* gene fusion in both tumors, which was subsequently confirmed by NUT-FISH and the monoclonal anti-NUT antibody. These cases represent a novel contribution to the spectrum of NUT-rearranged head and neck malignancies. This adnexal carcinoma variant should not be confused with the highly lethal NUT carcinoma based on NUT immunoreactivity alone.

## Introduction

Gene rearrangements involving the *NUT Midline Carcinoma Family Member 1 (NUTM1)* gene (also known as Nuclear Protein in Testis or *NUT*) mapped to chromosome 15q14 have emerged as a reliable genetic marker for a highly lethal poorly differentiated carcinoma with strong predilection for mediastinal and sinonasal midline structures, mainly of children and young adults [1, 2]. With increasing use of NUT immunohistochemistry (IHC) however, NUT carcinoma became more frequently identified over a wide patient’s age and in diverse anatomic sites including lateralized (non-midline) organs such as kidney, major salivary glands, extremity soft tissue and others [3–5]. This has led to a wider inclusion of the NUT IHC in the panel used for classifying undifferentiated or unclassified neoplasms irrespective of site. In the vast majority of cases, the recurrent genomic translocation t(15;19) (q13;p13.1) results in fusion of *NUTM1* to the gene *Bromodomain Containing 4* (*BRD4*), a member of the bromodomain gene family in 78% of cases [2, 6]. Rarely, other fusion partners have been described, including *BRD3* (15%) as well as non-bromodomain family members like *NSD3* (6%) and *ZNF532* (< 1%) [2, 6].

Almost one half of all NUT carcinoma cases are located in head and neck sites [7]. Sinonasal cavities harbor half of the head and neck cases with the remainder distributed between other organs including the larynx, salivary glands and other exceedingly rare locations [4, 7–9]. We herein describe two cases of auditory canal porocarcinomas carrying a *YAP1-NUTM1* translocation. Given the young age of one of the patients and hence the risk for misclassification of his tumor as an aggressive NUT carcinoma on the basis of NUT immunoexpression alone, we believe reporting these cases is of utmost relevance for the differential diagnosis of NUT-positive neoplasms in head and neck routine surgical pathology practice.

## Materials and Methods

The two cases were identified prospectively in our routine files. Case one (index case) was initially interpreted as unusual adnexal-type carcinoma on biopsy and due to the young age and unusual site was sent for RNA fusion testing to rule out translocations. This index case was diagnosed shortly before the recent publication by Sekine et al. who described high frequency of *NUTM1* rearrangements in poroma and porocarcinoma of the skin [10]. NUT immunohistochemistry (IHC) was performed following the molecular results. Case two was diagnosed as porocarcinoma based on its identity with the index case and NUT IHC was performed directly during routine evaluation. The tissue specimens were fixed in formalin and processed routinely for histopathology. IHC was performed on 3-µm sections cut from paraffin blocks using a fully automated system (“Benchmark XT System”, Ventana Medical Systems Inc., 1910 Innovation Park Drive, Tucson, Arizona, USA) and the following antibodies: pankeratin (clone AE1/AE3, 1:40, Zytomed, Berlin, Germany), CK5 (clone XM26, 1: 50, Zytomed), CK7 (OV-TL, 1:1000, Biogenex), p63 (SSI6, 1: 100, DCS), S100 protein (polyclonal, 1:2500, Dako), SOX10 (polyclonal, 1:25, DCS), synaptophysin (clone SY38, 1:50, Dako), (polyclonal rabbit antibody, 1:100, Dako), and anti-NUT antibody (clone C52B1, 1:45, Cell Signaling). Interpretation of results of the NUT IHC was based on published data showing distinctive nuclear immunoreactivity limited to the neoplastic cells. Normal testicular tissue was used as a positive control [3]. As a control, a cohort of eleven other carcinomas of the auditory canal were tested for NUT expression.

Mismatch repair (MMR) deficiency was assessed using immunohistochemical staining for MLH1 (clone ES05, 1:50, Dako), PMS2 (clone EP51, 1:40, Dako), MSH2 (clone G2-19-1129, prediluted, Ventana), and MSH6 (clone MSH6, 1:300, BD Pharmingen). Unequivocal nuclear staining in the tumor cells was considered retained (normal) expression. Normal cells in the background stroma served as internal control.

### Next Generation Sequencing and FISH Testing

Both tumors were tested for gene fusions using the RNA panel. In addition, the index case was also tested for gene mutations using the TST170 DNA gene panel. RNA was isolated from formalin-fixed paraffin embedded (FFPE) tissue sections using RNeasy FFPE Kit of Qiagen (Hilden, Germany) and quantified spectrophotometrically using NanoDrop-1000 (Waltham, United States). Molecular analysis was performed using the TruSight RNA Fusion panel (Illumina, Inc., San Diego, ca., USA) with 500 ng RNA as input according to the manufacturer's protocol. Libraries were sequenced on a MiSeq (Illumina) with > 3 million reads per case, and sequences were analyzed using the RNA-Seq Alignment workflow, version 2.0.1 (Illumina). The Integrative Genomics Viewer (IGV), version 2.2.13 (Broad Institute, REF) was used for data visualization.

To analyze the mutational status of commonly cancer related genes, DNA was isolated from FFPE tissue sections using the Maxwell 16LEV Blood DNA kit (Promega, Madison, USA) and submitted to hybrid-capture enrichment-based sequencing analysis using the TruSight Tumor 170 (TST170) gene panel (Illumina) according to the manufacturer`s protocol. Libraries were sequenced on a Next Seq550 (Illumina) and analyzed for single nucleotide mutations, insertions, deletions and copy number variations using the TruSight Tumor 170 software (BaseSpace Sequence Hub, Illumina) with human genome hg19 as reference.

Fluorescence in-situ hybridization (FISH) was performed using the ZytoLight *SPEC NUT* Dual Color Break Apart Probe (ZytoVision, Bremerhaven, Germany) with standard protocols according to the manufacturer`s instructions. Fifty tumor cells were visually inspected using a fluorescence microscope. The presence of two pairs of fused green and orange signals was considered normal findings. On the other hand, presence of one fused orange/green signal and one separate orange and green signals indicates translocation.

## Results

### Clinical History

#### Case 1

**Fig. 1:**
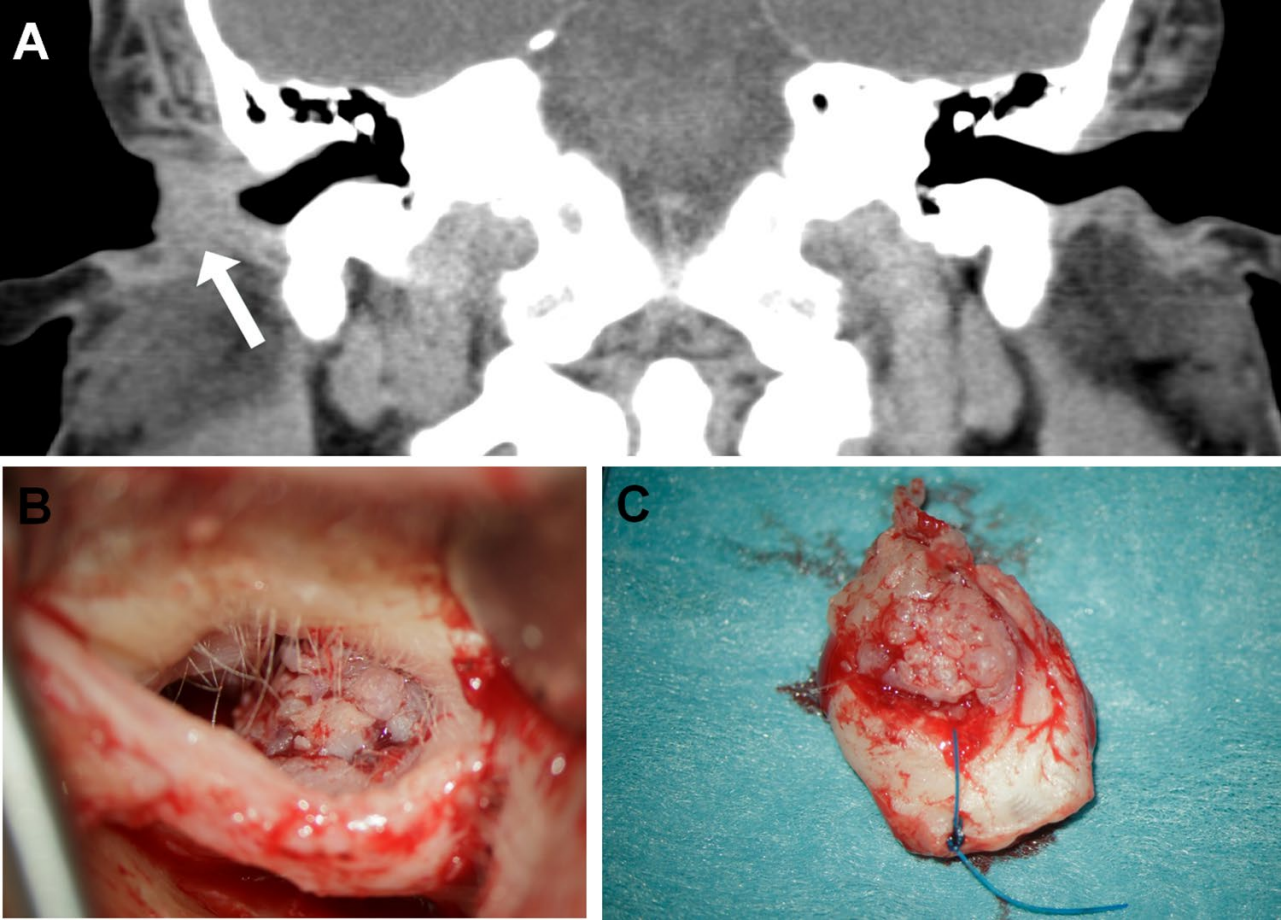
Representative examples of the appearance of the *YAP1-NUTM1*-rearranged porocarcinoma of the auditory canal on CT (**a** Case 1) and at surgery (**b** Case 2). The excised tumor in Case 2 showed a well circumscribed flat-exophytic to warty lesion (**c**)

A 29 year-old patient without any significant past medical or family history presented with a slowly growing lesion in the external auditory canal of 24 months duration (Fig. 1a). The patient did not attend a first appointment for biopsy, which was obtained 7 months later. There was no evidence of regional lymphadenopathy or distant metastasis. Following complete local excision of the tumor, he received adjuvant radiotherapy. Currently, the patient is alive without recurrence or metastases (12 months).

### Case 2

A 82 year-old man presented with an incidentally found tumor in the right outer ear canal during a routine check-up by his ENT specialist. The patient had no complaints concerning the tumor. In the preoperative CT-scan, a tumor was visible in the outer ear canal with slight bony erosion. There was no sign of regional lymphadenopathy. The tumor was locally excised under the strong suspicion of a carcinoma (Fig. 1b, c). Intraoperative frozen section suggested the diagnosis of porocarcinoma which was then confirmed on permanent sections. No adjuvant treatment was given. The patient is alive and well 6 months after surgery.

### Pathological Findings

**Fig. 2:**
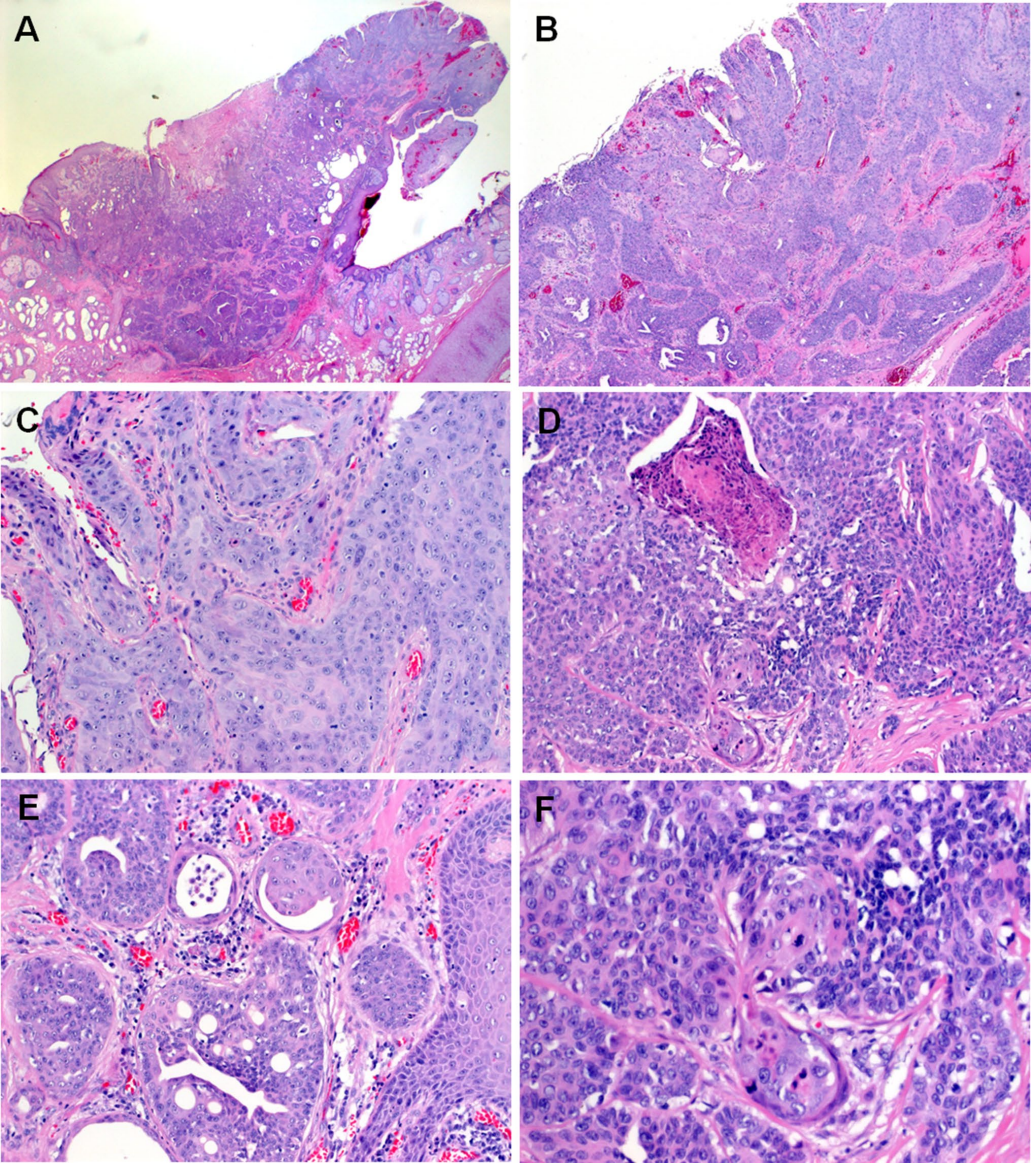
Representative images of Case 1. **a** Whole mount section of Case 1. **b** Closely juxtaposed squamous (upper right) and ductal (lower left) components are seen. **c** Higher magnification of the squamous component. **d** Abrupt keratinization is seen focally. **e** Prominent ductal differentiation with pseudocribriforming. **f** Prominent poroid monomorphic cells interrupted by squamous islands with mitosis (midfield)

Histological features of the two tumors were so strikingly similar that the second case which presented a few months after the first one was interpreted at frozen section as consistent with porocarcinoma. The tumors measured 1.5 and 0.8 cm, respectively. They presented as small polypoid protrusions of the skin with well-defined margins (Fig. 1b, c). Histological examination showed an infiltrating epithelial neoplasm composed of monomorphic atypical medium-sized polygonal to elongated epithelial cells with oval or elongated nuclei, distinctive small nucleoli and a moderate rim of pale-eosinophilic cytoplasm arranged predominantly in well-circumscribed solid nests surrounded by delicate fibrous stroma. Some of the nests showed foci of central necrosis. Other areas showed variable ductal differentiation with prominent microcystic glands with focal pseudocribriforming. The neoplasms merged with a superficial frankly squamous tumor component with higher degree of atypia and associated surface ulceration (Fig. 2). In some areas, the gland-like cystic structures were reminiscent of ceruminal gland lesions or suggestive of a pre-existing benign cystic component. Case two showed prominent involvement of the sweat ducts approaching their superficial openings on top of the epidermis. Here some superficial lateral spreading with replacement of the epidermis to a variable extend was observed. The mitotic activity was low (< 4 mitoses/10 high power fields). Foci of abrupt adnexal-type keratinization were seen as well. Representative examples of the two tumors are depicted in Figs. 2 (Case 1) and 3 (Case 2).

Immunohistochemistry showed diffuse expression of pankeratin AE1/AE3, CK5 (Fig. 4a), p63 (Fig. 4b) and SOX10 (Fig. 4c). Diffuse aberrant expression of TP53 was seen in both cases (Fig. 4d). S100, androgen receptor and synaptophysin were negative. CK7 highlighted focal ductal cells. Based on focal antipolar trichilemmal-like nuclear features in Case 1, the mismatch repair proteins MLH1, MSH2, MSH6 and PMS2 were stained; all showed retained physiological nuclear expression (data not shown). The NUT IHC showed uniform strong granular punctate to diffuse nuclear staining in all of the neoplastic cells. This staining highlighted the neoplastic cells in areas with reactive-like bland epithelial glands and also stained the frankly squamous superficial component, confirming their neoplastic origin (Fig. 4e–g).

**Fig. 3:**
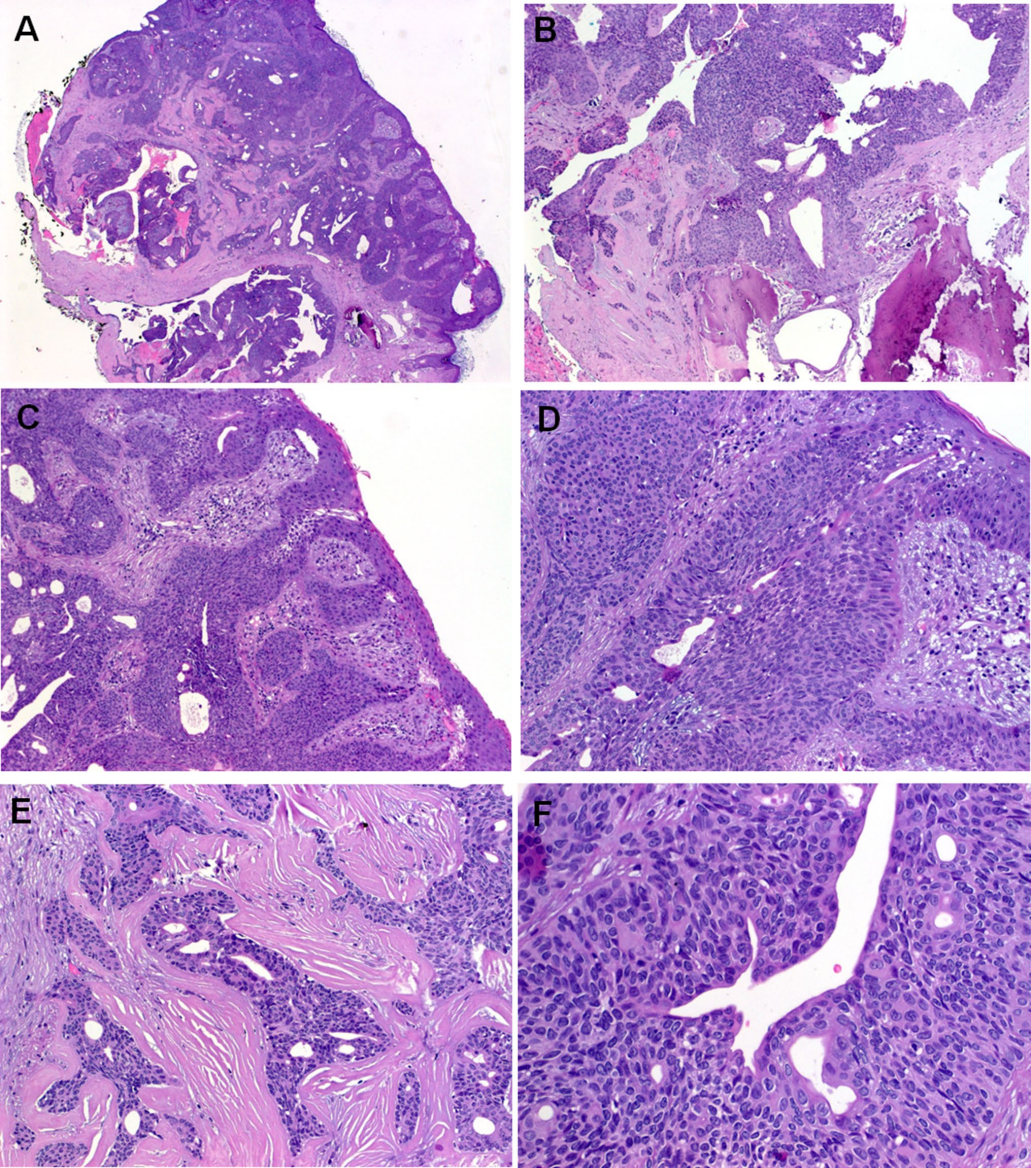
Representative images of Case 2. **a** Whole mount shows basaloid neoplasm with prominent ductal and cystic areas. **b** Focal erosion of underlying bone is seen. **c** Prominent replacement of the normal ducts up to the epidermal openings is seen, note lateral spreading replacing the epidermis to variable extent. **d** Higher magnification of **c**. **e** Prominent sclerosis was seen in purely ductal areas. **f** Solid aggregates of monomorphic basaloid poroid cells are seen surrounding central ducts

**Fig. 4:**
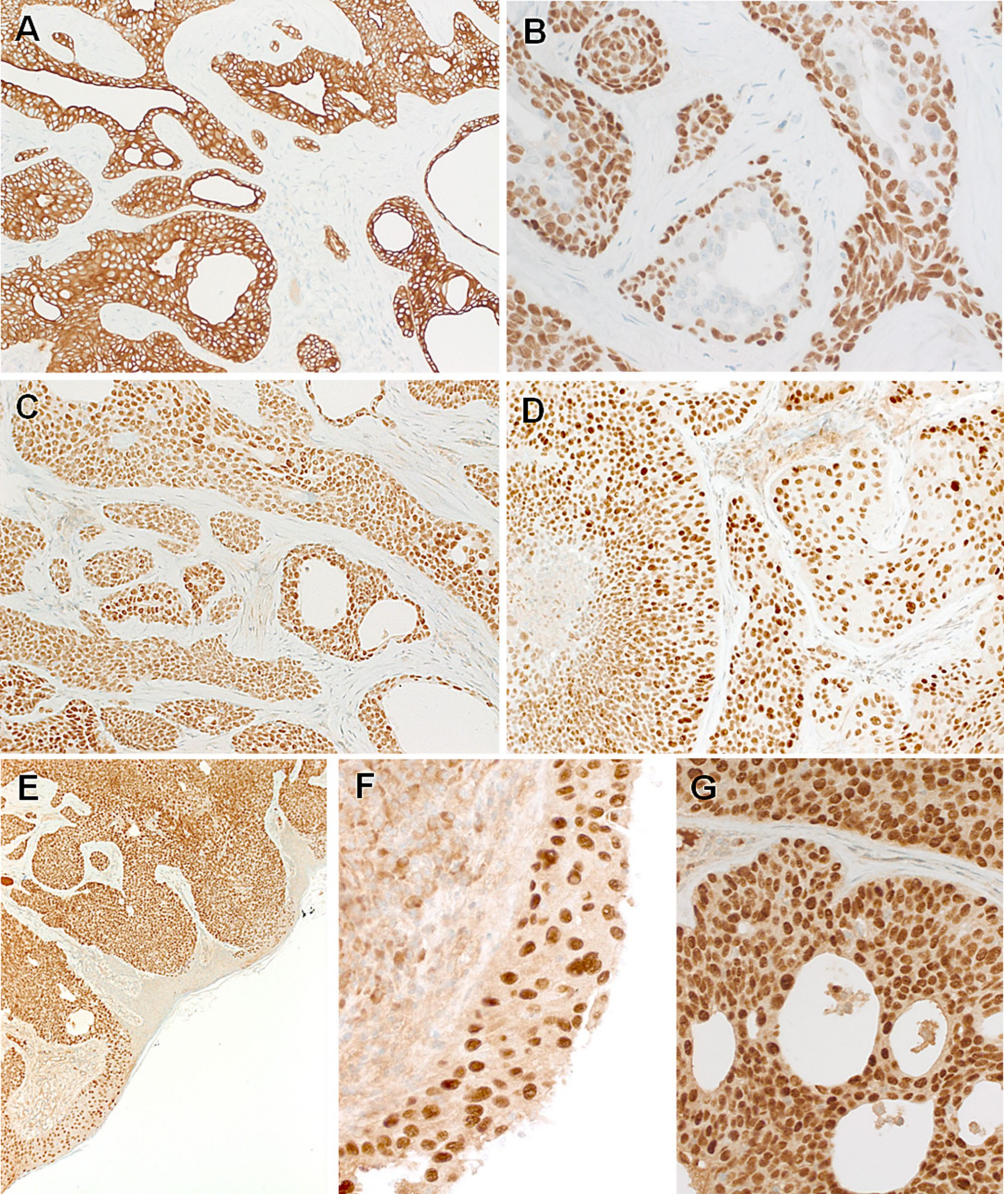
Representative images of the immunohistochemistry. Both tumors are strongly positive for CK5 (**a**) and p63 (**b**), note focal sparing of ductal cells in the p63 stain (**b**). SOX10 is expressed in all cells of both tumors (**c**). Aberrant TP53 is observed in both cases (**d**). Diffuse NUT expression is seen and it highlights the lateral spreading along the epidermis sparing non-involved epidermal cells (**e**, **f**). At high-power, the NUT reactivity is distinctly nuclear (**g**)

### Molecular Findings

**Fig. 5:**
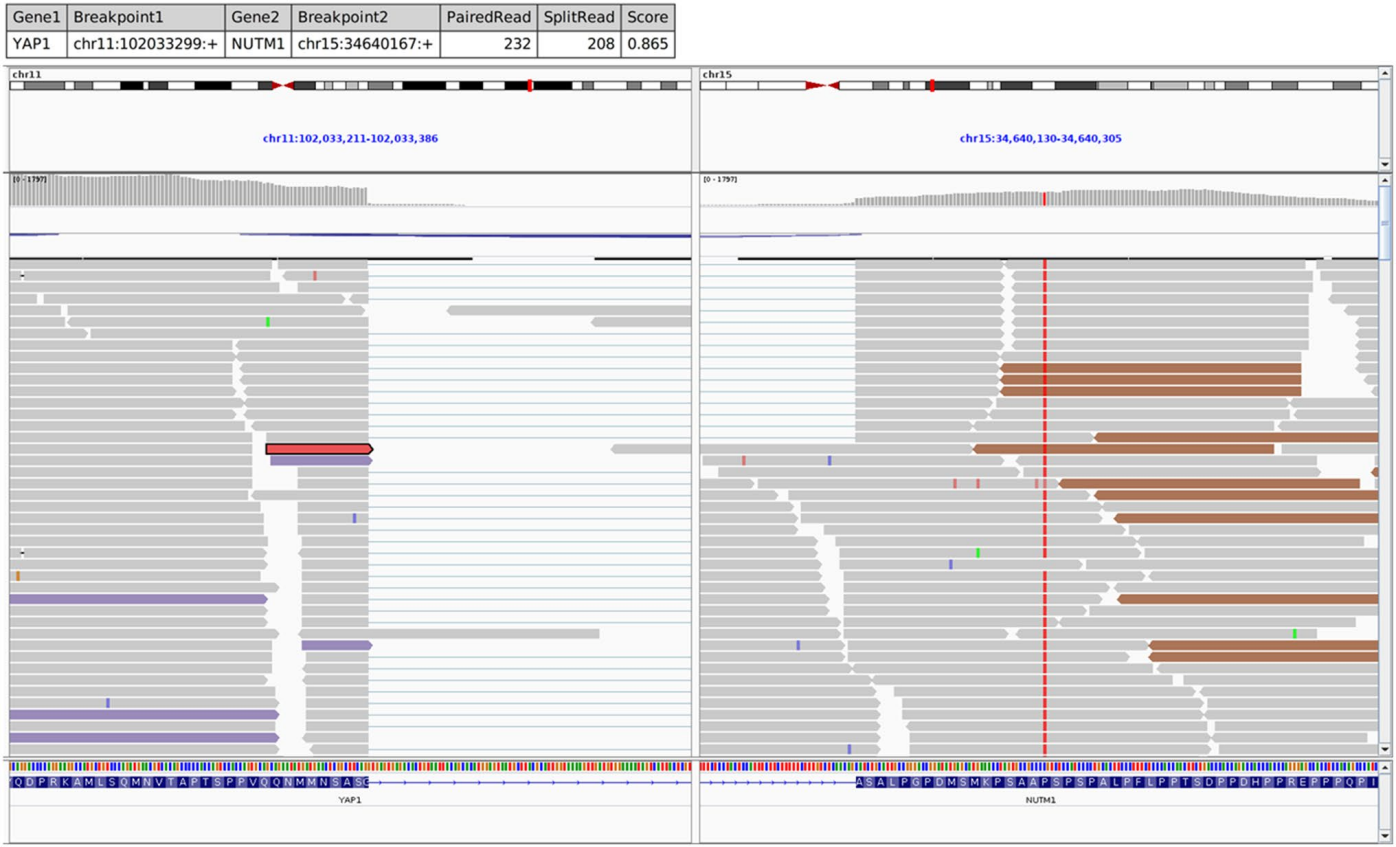
IGV split-screen view of read alignments of the identified YAP1-NUTM1 Fusion event. Shown are the breakpoints in the YAP1 locus (left) and the NUTM1 locus (right), respectively. Alignments whose mate pairs are mapped to the fusion sequence on the other chromosome are color-coded. The purple colored alignments on the left panel and the brownish alignments on the right are mate-pairs, illustrating the fusion event. All other alignments are colored grey.

The NGS RNA fusion analysis revealed the *YAP1-NUTM1* gene fusion in both tumors, in which exons 1–3 of the *YAP1* gene were fused *in frame* to exon 2 of the *NUTM1* gene (Fig. 5). FISH testing confirmed the *NUTM1* gene locus rearrangement in most of the neoplastic cell nuclei (not shown). Furthermore, a mutational screen using a targeted panel of commonly mutated, cancer related genes was performed in Case 1. This analysis revealed no additional mutations, consistent with the role of the *YAP1-NUTM1* fusion as sole oncogenic driver mutation in the tumor.

### Control Cohort

The control cohort (n = 11) of auditory canal carcinomas of different histological types retrieved from our routine files included six squamous cell carcinomas (SCC; five non-keratinizing and one keratinizing), two adenoid cystic carcinomas, two low-grade adenocarcinomas NOS and one basal cell carcinoma. None of these 11 carcinomas showed NUT expression by immunohistochemistry.

## Discussion

Poroma is a benign skin adnexal neoplasm showing terminal sweat gland duct differentiation [11]. Poromas are composed histologically of a variable combination of poroid cells and so-called cuticular cells. The lesion may be confined to either the dermis or the epidermis, or involve both [11]. Variable ductal differentiation is observed [11]. Porocarcinoma is considered the malignant counterpart of poroma and may arise either de-novo or from pre-existing benign poroma [12]. It typically presents as an ulcerated nodular lesion with a very wide age range (12–91 years) [12, 13]. The lower extremities are mainly affected, but other sites including the head and neck may be affected as well [12, 13]. Although focal squamous differentiation is considered rare in porocarcinoma, a predominantly squamous variant has been proposed [14]. A small subset of cases is strictly intradermal (so-called porocarcinoma in-situ).

The current cases illustrate examples of porocarcinoma presenting at unusual or unexpected sites. During preparation of this case report, a seminal study reported high frequency of *YAP1-NUTM1* gene fusions in subsets of porocarcinoma and poromas [10].

The first case presented herein represents a true challenge due to the unusual age of the patient, unusual presentation and the difficulty and pitfalls associated with interpretation of the molecular results. The poroid nature of this case was not recognized during initial assessment of the preoperative biopsy so that a descriptive diagnosis of adnexal carcinoma was made. As part of our practice to submit unusual and unclassified neoplasms for molecular testing, we sent this case in the setting of initial assessment to our molecular laboratory where the NGS RNA panel revealed the *YAP1-NUTM1* gene fusion. Notably, our diagnosis before and after detection of the *NUTM1* gene fusion was concordantly that of an unusual type of malignant skin adnexal neoplasm (adnexal carcinoma) of the auditory canal. This was mainly based on the relatively monomorphic squamoid neoplastic epithelial cells with prominent antipolar nuclear alignment reminiscent of the trichilemmal epithelium. As the study by Sekine et al. [10] was not published at the time of reporting Case 1, we initially considered this unusual genetic alteration of uncertain significance. Accordingly, we emphasized the clear-cut difference between our case and conventional NUT carcinoma in the pathology report to avoid overtreatment [1, 2, 6]. Strong and specific nuclear NUT expression confirmed the genetic finding and is in line with an oncogenically active *NUTM1* fusion.

Using RNA sequencing and reverse transcription PCR, Sekine et al. identified high frequency of *YAP1-MAML2* and *YAP1-NUTM1* fusions in poromas (92/104 cases; 88.5%) and porocarcinomas (7/11 cases; 63.6%) [10]. One poroma harbored a *WWTR1-NUTM1* gene fusion. YAP1 and WWTR1 are involved in the regulation of the Hippo signaling pathway via their interactions with TEAD [15]. The tumorigenic effect of YAP1 has been verified in recent studies [15]. Therapeutic opportunities targeting the YAP1/TEAD-dependent transcription are being tested in different neoplasms [16].

These recent observations underline the need for careful interpretation of immunohistochemical and molecular results in this era of rapidly evolving molecular pathology. Given that the NUT IHC is being increasingly part of the workup of any unusual or unclassified neoplasm, especially by head and neck pathologists, there is a risk to misinterpret such unexpected NUT expression as indicative of NUT carcinoma. Inappropriate diagnosis of NUT carcinoma would be associated with significant impact on patient’s treatment and prognosis. Furthermore, detection of *NUTM1* rearrangements in benign poromas as well precludes the notion that the *NUTM1* gene is a lethal fusion partner in neoplasms and underlines the evolving and continuous role of standard histology and phenotyping in diagnosis and classification of neoplasms.

It is remarkable that both tumors in our study revealed strong and diffuse expression of SOX10, suggesting that SOX10 might be of value in distinguishing porocarcinoma from squamous cell carcinoma on limited biopsies. However, the sparsity of data on SOX10 expression in poroid neoplasms precludes any definitive statement. Cassarino et al. did not detect SOX10 reactivity in 12 tested poromas [17]. However, another study by Lezcano et al. detected SOX10 expression in one of 17 poromas and in one of 4 porocarcinomas [18]. Both studies used same mouse monoclonal anti-SOX10 antibody (clone BC34: Biocare Medical; Concord, California) [17, 18] and we used a polyclonal one. Whether this discrepancy is related to the sensitivity and specificity of the antibodies used in different studies or is influenced by the genotype, remains an issue of future studies comparing different antibodies on genetically characterized poroid neoplasms. Indeed, we observed similar strong SOX10 expression in a recent case of *YAP1-NUTM1*-rearranged benign skin poroma of the upper extremity suggesting that the antibody we use is more sensitive in detecting SOX10 in poroid neoplasms.

In summary, we describe herein the first two cases of auditory canal porocarcinoma harboring a *YAP1-NUTM1* gene fusion. Recognition of this entity is mandatory to avoid misinterpretation as lethal NUT carcinoma. These cases add to the expanding spectrum of *NUTM1*-rerranged neoplasia of the head and neck.
